# Informing Scalable Implementation Strategies for Returning Individual Research Results: A Mixed-Methods Study Across Multisite Research Teams

**DOI:** 10.21203/rs.3.rs-9621011/v1

**Published:** 2026-05-25

**Authors:** Denise Kent, Michelle Villegas-Downs, Mary Pasquinelli, Adriana Arcia, Lauren Greene, Sarah Darski, Elizabeth Sugar, Ravi Kalhan, Subidsa Srikantha, Hugh Musick, Jerry Krishnan, Lynn Gerald

**Affiliations:** University of Illinois at Chicago; University of Illinois at Chicago; University of Illinois at Chicago; University of San Diego; University of Illinois at Chicago; University of Illinois at Chicago; Johns Hopkins University; Northwestern University; University of Illinois at Chicago; University of Illinois at Chicago; University of Illinois at Chicago; University of Illinois at Chicago

**Keywords:** Implementation science, Consolidated Framework for Implementation Research, Individual research results (IRRs), Return of results, Multisite research, Mixed-methods, Health communication, Participant engagement, Incidental findings, Implementation strategies

## Abstract

**Introduction:**

Despite growing national guidance supporting the return of individual research results (IRRs) to participants, scalable, theory-informed implementation strategies to support consistent and effective return across multi-site studies remain underdeveloped. This study aimed to identify multilevel determinants of IRR return and to use those determinants to inform the inform the specification of implementation strategies for multisite research settings.

**Methods:**

A mixed-methods study, guided by the Consolidated Framework for Implementation Research (CFIR), was conducted within the American Lung Association’s NIH-funded Lung Health Cohort (LHC) study, a 26-site multisite research network. A structured CFIR-informed survey and qualitative thematic analysis were used to examine IRR return practices. Survey and focus group questions were mapped to CFIR domains. Descriptive statistics summarized quantitative findings, and inductive thematic analysis was applied to open-ended survey responses and focus group data. Emergent themes were mapped to CFIR domains and synthesized to inform implementation strategy development.

**Results:**

Fifty-one research team members representing all twenty-six sites participated in a structured survey. Multilevel determinants influencing IRR return were identified across all CFIR domains. Two focus groups (n = 26) were held with research coordinators and study investigators to understand key challenges including complexity of communicating findings of uncertain clinical significance (Innovation), variability in participant access to care and health literacy (Outer Setting), heterogeneity in workflows and role delineation (Inner Setting), differences in team confidence and preparedness (Characteristics of Individuals), and lack of standardized communication tools and monitoring systems (Implementation Process). These determinants were used to specify a set of theory-informed implementation strategies, including standardized communication templates, role-specific scripts, clinical escalation pathways, participant navigation supports, and workflow tracking systems.

**Conclusions:**

This study moves beyond descriptive assessment to inform the development of scalable, theory-informed implementation strategies for returning IRRs in multisite research. By linking empirically identified determinants to actionable strategies, these findings provide a foundation for future intervention development and evaluation aimed at improving consistency, efficiency, and participant-centered communication of research results.

## Background

Returning Individual Research Results (IRRs) to participants in biomedical research is increasingly recognized as an essential component of participant-centered research. Disclosing individual results acknowledges participants’ contributions, promotes transparency, and strengthens trust between research teams and the communities they serve^1–3^. However, in large multisite studies, implementing the return of IRRs requires more than a commitment; it demands clear workflows, defined roles, institutional infrastructure, and team preparedness.

Although national guidance encourages the return of valid and actionable findings^2,4^, there is limited empirical evidence describing how research teams operationalize this process in real-world, multisite settings. Practical questions remain regarding what types of results are returned, how communication responsibilities are distributed across team members, how variability across institutions influences implementation, and what support research teams need to communicate results effectively. These operational realities are particularly salient in studies generating imaging, laboratory, and physiologic data, where findings may vary in clinical clarity and urgency.

The National Institutes of Health-funded American Lung Association Lung Health Cohort (LHC) study is a multisite cohort study collecting chest computed tomography (CT), laboratory, and spirometry data from healthy young adults. While the LHC protocol generates clinically relevant findings, the processes by which results are returned to participants vary across sites, creating potential inconsistencies in communication workflows, team preparedness, and participant follow-up pathways. Understanding these implementation challenges is critical for developing scalable, standardized approaches that remain adaptable to local site contexts.

The American Lung Association-funded REturning Computed tomography rEsults to actIVE Lung Health Cohort participants (RECEIVE) was designed to systematically examine how IRRs are returned within the LHC network. Guided by the Consolidated Framework for Implementation Research (CFIR), this study sought to (1) characterize current practices for returning IRRs across participating sites, (2) identify barriers and facilitators at the organizational, individual, and process levels, and (3) generate practical recommendations to inform the development of standardized yet flexible communication tools for large research networks.

In this report, we focus on findings from the LHC research team members, including coordinators, nurses, and investigators, to understand the implementation realities of returning IRRs in a multisite cohort. Beyond characterizing current practices, this study informs the development of implementation strategies to support scalable, consistent, and participant-centered return of IRRs. By identifying modifiable determinants across domains of the CFIR, we generate theory-informed strategies that can be operationalized in future intervention design and evaluation.

## Methods

**Figure 1:**
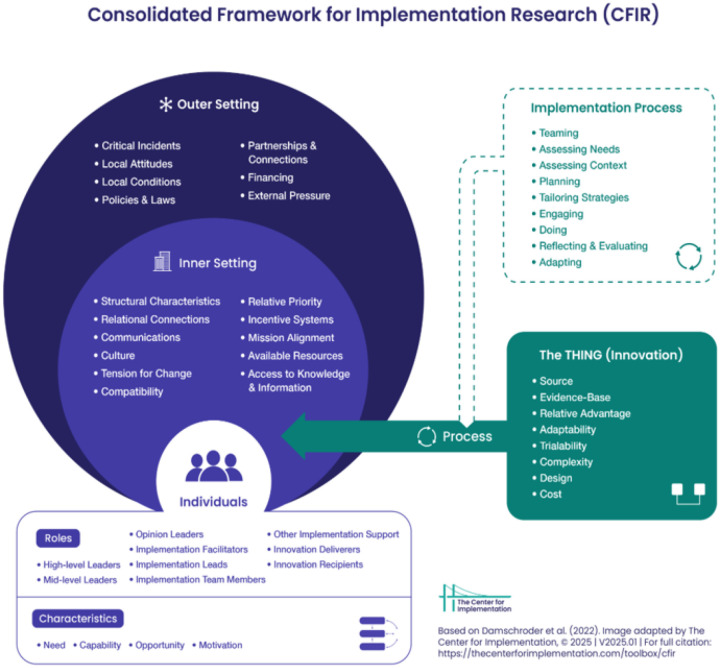
See image above for figure legend.

This study used a mixed-methods design and naturalistic inquiry^5^ to not only describe current return of results practices within the LHC, but to systematically inform the specification of implementation strategies aligned with identified CFIR-based determinants. A structured survey, guided by CFIR^6^, was developed to capture research team perspectives. We selected CFIR because returning IRRs represents a complex implementation process occurring within healthcare-affiliated research settings. Although CFIR was originally developed to assess implementation of clinical interventions, its multilevel domains addressing organizational context, individual characteristics, workflow processes, and external influences are well suited to evaluating return of results research practices. In this study, CFIR provided a structured framework to examine variability in IRRs communication workflows, team preparedness, and institutional infrastructure across multisite settings (Fig. 1).

We followed the Standards for Reporting Qualitative Research (SRQR) (Supplement A)^7^. The University of Illinois Chicago Institutional Review Board approved and monitored the study (#2024 − 0782). All participants provided informed consent. Survey participation was voluntary, and participants received a $10 e-gift card as compensation for their time. Focus group participation was not compensated. All survey and focus group data were de-identified prior to analysis.

Focus group discussions were conducted by the principal investigator (DAK) with LHC research team members to further explore experiences, challenges, preferences, and recommendations related to the return of IRRs. These discussions provided in-depth qualitative insights, capturing nuances in research team viewpoints and surfacing contextual factors not easily identified through surveys alone.

### Participants:

Eligible respondents were LHC study research team members from any of the 26 participating research sites who were involved in reviewing or returning IRRs (including laboratory data, spirometry, and chest CT results) to participants. The research team included Principal Investigators (PIs), Co-Investigators, Study Coordinators, Research Nurses, and others. All LHC team members who completed the survey were invited to participate in focus group sessions held during an in-person steering committee meeting in Nashville, Tennessee (March 2025).

### Survey Development

A structured, confidential web-based survey was developed in consultation with an implementation scientist (LBG) and a survey design expert (HM). The items were informed by constructs from the CFIR framework^6^. Sample survey questions mapped to the CFIR model dimension^6^ include: 1. Innovation (What IRRs are being returned?), 2. Outer Setting (What supports are in place to return IRRs?), 3. Inner Setting (How can the LHC study team better support the return of IRRs?), 4. Characteristics of Individuals (Do you feel you have the answers to participant questions?), and 5. Implementation Process (What are your overall reflections?) (see Supplemental B). By grounding the survey in the CFIR framework, our team created items assessing current IRRs return procedures, perceived utility, barriers, and support needs. The LHC Data Coordinating Center shared a list of all LHC study team members, and those individuals were contacted by email between October and November of 2024 to complete the survey. The survey collected in REDCap included a mix of multiple-choice, Likert-scale, and open-ended questions (see Supplement B).

### Open-Ended and Focus Group Questions

Qualitative data was collected through items in the web-based survey (e.g., free text responses to survey items regarding benefits, challenges, and support needs associated with returning IRRs) (see Supplement B) and focus group discussion questions (see Supplement C). Focus group questions were also informed by the CFIR framework and designed to elicit deeper insights into multilevel implementation factors influencing the return of IRRs. Questions mapped to CFIR domains included: Innovation (e.g., What types of results are most challenging to communicate? Would templates or scripts would improve clarity?); Outer Setting (e.g., What barriers do participants face when following up on abnormal findings? What external resources are needed?); Inner Setting (e.g., Where are workflow gaps or protocol inconsistencies? How can clinical teams be better integrated?); Characteristics of Individuals (e.g., Do you feel adequately prepared to explain incidental findings? What training or expertise would increase confidence?); and Implementation Process (e.g., What challenges have emerged in practice, and how might workflows be standardized while maintaining site flexibility?). Focus groups sessions were organized by staff type (e.g., coordinator and investigator) and conducted by the principal investigator (DAK) and two members of the team (SD and LG). Two focus group sessions were held, one for LHC study coordinators on March 13, 2025, and the other for LHC study investigators on March 14, 2025. To minimize potential bias or influence on participant responses, participants were informed that feedback, both positive and critical, was welcomed and would not impact their roles within the LHC study. In the coordinator session, small-group discussions were conducted prior to large-group sharing to encourage open dialogue among peers. Due to logistical constraints during the in-person steering committee meeting, focus groups were not audio-recorded; instead, two members of the research team (SD and LG) independently documented detailed notes in real time. Both focus group sessions were conducted in-person in Nashville, Tennessee during a break from the ALA- ACRC steering committee meeting.

The coordinator focus group discussion was structured so that two questions were randomly given to 2–3 LHC study coordinators to discuss. Team members were given 15 minutes to discuss their assigned questions and record their responses on large post-it notes. After this discussion period, the RECEIVE research team (DAK, LG, SD) facilitated a group share-out, allowing each small group to present their responses to the questions they were given to all LHC study coordinators present. Other team members from the larger group were also invited to contribute their thoughts. The entire coordinator focus group session lasted 65 minutes.

The investigator focus group session took place during the lunch break at the ACRC meeting on March 14th. Investigators were invited to join one of two tables designated for the focus group discussion. The RECEIVE research investigators (DAK and LBG) facilitated the discussion using the investigator focus group questions. The Investigator focus group discussion lasted approximately 45 minutes.

Immediately following each focus group, the facilitators and note-takers met to reconcile and synthesize notes to ensure completeness and accuracy of captured content. Data saturation was considered achieved when participants were no longer generating new themes or substantive insights during discussion. This approach allowed for comprehensive documentation of key points while maintaining feasibility within the meeting setting.

### Data Analysis

Survey data were cleaned and analyzed descriptively by the research team (DAK, MVD, and SS) using summary statistics. Results included frequencies and proportions for categorical variables (e.g., types of results returned, communication methods, respondent roles), means and standard deviations for continuous variables (e.g., research team comfort scores), and cross-tabulation for role-specific practices.

Open-ended survey responses and detailed focus group notes were analyzed using thematic analysis by members of the research team (DAK, HM, and SS)^8^. Initial coding was conducted inductively to identify patterns emerging directly from the data rather than applying predetermined categories. Two investigators (DAK and HM) independently reviewed and coded the data line-by-line, generating preliminary codes reflecting recurrent ideas, barriers, and recommendations. The investigators met regularly to compare interpretations and resolve discrepancies through consensus.

Through an iterative analytic process, related codes were grouped into broader thematic categories based on conceptual similarity and relevance to the study aims. Major themes were defined as patterns that were either (1) recurrent across multiple respondents or sites and/or (2) described as substantively important to return-of-results implementation, even if less frequent. Themes were examined across respondent roles and sites to identify similarities and differences.

After themes were developed inductively, they were mapped to the five CFIR domains to provide an organizing implementation framework. Triangulation of survey and focus group data enhanced analytic credibility and supported cross-validation of findings. Representative verbatim quotes were selected to illustrate each major theme. The final thematic structure is presented in the Results section. As part of the analytic synthesis, the research team developed representative vignettes to integrate findings across CFIR domains and illustrate how multilevel implementation determinants manifest in real-world practice.

## Results

Fifty-one LHC study research team members representing all 26 sites completed the survey (39% response rate), and 26 team members participated in focus groups.

### Innovation: Characteristics of the IRRs Return Process

We examined the types of results returned, variability in disclosure practices, and the perceived complexity of communicating findings. Across sites, research teams reported returning chest CT, laboratory, and spirometry results. Abnormal findings were more consistently returned than normal findings (see Table 1; e.g., 96% of abnormal chest CT results vs. 81% of normal chest CT results). Although the LHC study protocol required review of clinically relevant abnormalities, sites varied in whether normal findings were routinely returned.

Focus group participants described difficulty communicating findings categorized as “abnormal but not clinically significant.” These findings, such as mild mosaic attenuation, small non-calcified nodules below threshold for action, or borderline laboratory abnormalities, were perceived as clinically low-risk but difficult to contextualize for participants. The team expressed concern that the term “abnormal” inherently triggered anxiety, even when no immediate follow-up was recommended. Coordinators in particular reported spending substantial time reassuring participants, clarifying that the finding did not indicate any follow-up but should be shared with their health care provider. Investigators similarly described tension between transparency and harm reduction, balancing transparency with concern about provoking unnecessary worry or prompting low-value follow-up care. These challenges increased perceived complexity of the return of results process and were frequently cited as a reason research team members desired standardized scripts, clearer language, and clinician-supported communication pathways (see Table 2). The clinical ambiguity of certain findings increased the perceived complexity of the return of results process.

Qualitative data provided important context for observed quantitative differences in comfort by role. Although overall comfort with returning IRRs was high, coordinators reported lower mean comfort scores than investigators. In focus groups and open-ended responses, coordinators described greater difficulty explaining uncertain imaging findings, managing follow-up logistics, and addressing participant questions without immediate clinical support. These narratives help explain role-based differences observed in survey responses and highlight how implementation challenges may vary across professional roles.

Participants reported that returning IRRs offered relative advantages, including early detection of health conditions (e.g., air trapping, coronary artery calcification) and strengthened participant engagement. However, the complexity of certain findings often required careful explanation and, in some cases, additional clinical support (see Table 2).

### Outer Setting: Participant Needs and External Context

Findings demonstrated that participant-level needs and external factors influence the return of IRRs across sites. Quantitative data indicated most sites relied on phone (92%) and HER-based communication (73%), research teams reported these modalities did not align with all participants’ (see Table 1). Some participants lacked primary care providers or insurance, complicating follow-up care when abnormal findings were identified.

Qualitative findings further described these gaps (see Table 2). Research team members described variability in participants’ access to technology, including limited use of patient portals, disconnected phone numbers, and inconsistent responsiveness to outreach attempts. These barriers often required repeated contact efforts and contributed to delays in successful result delivery. In addition, participants’ health literacy was identified as a key determinant influencing comprehension of results. Team members reported that technical or ambiguous language (e.g., abnormal not clinically significant), led to misunderstanding, requiring additional time for clarification and reassurance.

Beyond communication, external healthcare access constraints shaped the return of IRRs process. Participants without established primary care providers or insurance faced challenges in acting on abnormal findings, effectively shifting responsibility for care navigation to research staff. As one respondent noted, lack of access to follow-up care “creates difficulty in finding appropriate access to the follow-up needed,” highlighting how structural barriers extend the scope of IRR return beyond information delivery to include informal care coordination.

### Inner Setting: Organizational Infrastructure and Workflow

Institutional workflows, staffing models, and infrastructure varied across sites. While chest CT scans in the LHC study are obtained for research purposes, the protocol requires that all scans be interpreted by a certified radiologist and that any clinically significant or incidental findings be communicated to participants. Notification of the participant’s primary care physician is left to the discretion of the local site PI, and responsibility for arranging follow-up care rests with the participant. Although the protocol provides overarching guidance for returning IRRs, it does not standardize operational procedures. As a result, implementation varied substantially across sites, including differences in timing of result return, personnel responsible for disclosure, and coordination between radiology, laboratory services, and research teams (see Table 1). While most sites reported returning results within 10 days, workflow bottlenecks, radiology delays, and local institutional processes occasionally extended timelines.

Focus group discussions revealed inconsistencies in role clarity, with multiple team members involved in reviewing or communicating results. Coordinators reported operational inefficiencies, including delays in data integration and uncertainty regarding responsibility for follow-up communication. Some participants favored centralized approaches to streamline processes and improve consistency (see Table 2); however, others expressed concern that centralization could weaken site-level relationships and trust.

### Characteristics of Individuals: Knowledge, Comfort, and Self-Efficacy

The team’s comfort and preparedness varied by role. Most respondents reported feeling “often” or “always” prepared to answer participant questions (73%), and overall comfort with returning results was high (see Table 1). However, qualitative findings revealed that non-clinical team members, particularly coordinators, more often expressed discomfort explaining complex or ambiguous findings. Investigators reported higher mean comfort levels compared to coordinators.

Team members described the emotional burden of communicating abnormal findings and the uncertainty associated with incidental imaging results. Many recommended additional training, decision-support tools, and clearer scripts to enhance confidence and reduce ambiguity. These findings indicate that individual knowledge and self-efficacy influenced research results return practices.

### Implementation Process: Planning, Execution, and Standardization

Findings highlighted variability in planning, execution, and monitoring of research results workflows. Communication methods included phone calls (92%), EHR messaging (73%), in-person visits (54%), and mailed letters (42%) (see Table 1). However, no standardized scripts or templates were consistently used across all sites. Team members reported that participants sometimes received results before the team were able to provide contextual explanation, contributing to confusion and anxiety.

Across domains, identified determinants were mapped to potential implementation strategies. For example, communication complexity and variability (Innovation, Inner Setting) informed the need for standardized templates and scripts; gaps in participant access and follow-up resources (Outer Setting) informed navigation and referral supports; and role ambiguity and variable confidence (Inner Setting, Characteristics of Individuals) informed role delineation and training strategies (see Table 2). These cross-domain linkages provide a foundation for structured implementation strategy development.

## Discussion

This study advances the field by moving beyond descriptive characterization of IRR return practices to the development of theory-informed implementation strategies. Using CFIR as a guiding framework, we identified multilevel, modifiable barriers and linked those to potential solutions to support scalable implementation of IRR return across multi-site research settings (Table 3).

To synthesize findings across domains, the research team developed a representative vignette (Table 4 Vignette A) that integrates multilevel determinants of IRR return within a single, real-world scenario. Rather than serving as a standalone illustration, the vignette functions as an analytic tool, demonstrating how barriers identified across CFIR domains co-occur and interact during routine implementation. The experience of the coordinator [“Maria”] reflects the convergence of challenges related to communication complexity (Innovation), participant context and resource limitations (Outer Setting), workflow variability (Inner Setting), role-based confidence (Characteristics of Individuals), and lack of standardized processes (Implementation Process).

Within the Innovation domain, findings highlight the complexity of communicating imaging and laboratory results, particularly those categorized as “abnormal but not clinically significant.” Across sites, these findings were consistently described as difficult to contextualize in a way that is both accurate and non-alarming. The vignette illustrates how this ambiguity is operationalized in practice, requiring coordinators to translate uncertain clinical information without standardized language or decision support. This aligns with prior literature on incidental findings, which emphasizes the risk of patient anxiety and downstream overutilization when results lack clear interpretive frameworks.^9,10^

The Outer Setting is reflected in the vignette through participant-level factors that shape communication and follow-up. Variability in health literacy, access to primary care, and familiarity with electronic health record systems influenced how results were received and acted upon. In the vignette, the absence of a primary care provider transforms a routine disclosure into a broader navigation challenge, underscoring how social and structural determinants extend the scope of IRR return beyond information delivery. These findings reinforce prior work demonstrating that return-of-results processes must account for access to care and support systems, particularly in populations at risk for disparities.^11,12^

Within the Inner Setting, institutional variability in workflows and role delineation contributes to inconsistent implementation. As depicted in the vignette, Maria navigates unclear responsibilities, fragmented communication pathways, and delays in data availability. Although overarching protocol guidance exists, the absence of standardized operational procedures results in site-level adaptation that can introduce inefficiencies and variability. This finding is consistent with prior multisite research demonstrating that protocol-level requirements without implementation specificity may inadvertently produce heterogeneity in practice.^13,14^

The Characteristics of Individuals domain is reflected in role-based differences in comfort, knowledge, and self-efficacy. While survey findings suggested overall confidence in returning results, qualitative data revealed that coordinators, particularly those without clinical training, experienced uncertainty when explaining ambiguous findings and managing participant questions. The vignette captures this tension, highlighting the cognitive and emotional burden associated with navigating the boundary between research communication and clinical interpretation. These findings extend concerns regarding therapeutic misconception and reinforce the need for structured supports to guide non-clinician communication of clinically relevant information.^1,15^

Finally, within the Implementation Process domain, the absence of standardized workflows, communication tools, and monitoring systems contributes to reactive and inconsistent practices. The vignette illustrates how participants may access results prior to receiving explanation, leading to confusion and increased anxiety. Without formal tracking systems or defined escalation pathways, coordinators rely on ad hoc processes to manage communication and follow-up. These findings align with national recommendations calling for systematic approaches to returning research results.^2^

To complement this analysis, a second vignette (Table 4 Vignette B) was developed to represent a future-state scenario in which CFIR-informed implementation strategies are applied. This vignette operationalizes potential solutions identified across domains, including standardized communication tools (Innovation), structured referral and navigation supports (Outer Setting), defined workflows and roles (Inner Setting), enhanced training and clinical backup (Characteristics of Individuals), and integrated tracking and monitoring systems (Implementation Process).

Taken together, the paired vignettes extend the CFIR analysis by illustrating not only the presence of multilevel barriers, but also how targeted, theory-informed strategies can reshape the implementation environment. This approach highlights that returning IRRs in multisite research is not a discrete task but a dynamic, multilevel process requiring coordinated intervention across domains. By embedding these insights within narrative form, the study advances understanding of how implementation determinants are experienced in practice and provides a foundation for designing scalable, participant-centered return-of-results systems.

### Limitations

Several limitations warrant consideration. This study relied on self-reported practices and perceptions, which may not fully reflect actual workflows or participant experiences. Although the survey included respondents from all 26 sites, the 39% response rate and voluntary participation may introduce selection bias, as individuals more engaged in returning results may have been more likely to respond. Focus groups were conducted during an in-person steering committee meeting, potentially limiting representation from individuals unable to attend.

Focus groups were facilitated by the principal investigator, who also serves as an investigator within the LHC study network. While structured discussion guides, small peer-based group discussions, and independent documentation were used to encourage candid dialogue and reduce facilitator influence, participants may have moderated their responses due to perceived power differentials. Social desirability bias within a collaborative research network context should therefore be considered when interpreting findings.^16^ Additionally, this data reflects the research team’s perspectives within a well-resourced clinical research network and may not generalize to other settings.

### Implications

The findings provide empirical insight into the operational realities of returning IRRs in multisite research. Our results support the development of adaptable yet standardized communication tools that can accommodate site-level variation. The RECEIVE team is currently surveying two additional stakeholders, the LHC study research participants and their healthcare providers. Insights across all stakeholders within the LHC study will guide the development of a return-of-results toolkit. Core components of the toolkit based on the data provided by the research team will include: (1) standardized participant-facing communication templates tailored by result type, (2) role-specific scripts and decision-support tools, (3) structured clinical escalation pathways for complex findings, and (4) workflow monitoring systems to track return of results and ensure timely communication. Future work should evaluate whether such tools improve the team’s confidence, workflow efficiency, and participant understanding.

Importantly, these findings also have implications for research funding structures. Returning IRRs in a timely, participant-centered manner requires dedicated personnel time, clinical review infrastructure, communication tools, and workflow monitoring systems, resources that are often not fully accounted for in traditional research budgets. Funding agencies should consider requiring and supporting explicit plans for real-time dissemination of individual research results, particularly in studies generating clinically relevant imaging or laboratory data. Embedding financial support for IRR return within research awards may strengthen recruitment and retention by reinforcing transparency and participant engagement, while also promoting ethical stewardship of participant-generated data.^2^ Future funding mechanisms should recognize IRR dissemination as a core component of study design rather than an ancillary activity.

## Conclusions

Returning IRRs has become a growing priority in participant-centered research, reflecting a commitment to transparency, trust, and engagement. However, many study teams encounter significant challenges that hinder effective communication of research results to participants. Overcoming these barriers will require harmonized protocols, sustained investment in the research team and infrastructure, and a steadfast commitment to addressing the needs of diverse participant communities. This work offers practical insights and establishes a foundation for best practices in returning IRRs, paving the way for continued progress and innovation in this evolving area of research.

## Supplementary Material

Supplementary Files

This is a list of supplementary files associated with this preprint. Click to download.

- SupplementsKent.docx

## Figures and Tables

**Table 1:** Survey Responses

Site	Total n (%)
**Type of Results Returned (n = 26)** *	
**Chest CT Results**	
Abnormal	25 (96)
Normal	21 (81)
**Lab**	
Abnormal	24 (92)
Normal	21 (81)
**Spirometry**	
Abnormal	23 (88)
Normal	19 (73)
**Method for Returning Results**	
Phone Call	24 (92)
Electronic Health Record (EHR)	19 (73)
During Follow-Up Visit	15 (58)
In-Person	14 (54)
Mail	11 (42)
Fax	1 (4)
Other	4 (15)
Respondent	Total n (%)
Role	(n = 51)
Coordinator	26 (51)
Research Nurse	3 (6)
Principal Investigator	16 (31)
Co-Investigator	4 (8)
Other	2 (4)
**Timing for Return of Results**	
**Chest CT Results**	
Within 10 days of the results being available	44 (86)
Between 11–30 days from the results being available	6 (12)
30 days or more	0 (0)
Results are Not Returned	0 (0)
Do not remember	0 (0)
No Response	1 (2)
**Lab**	
Within 10 days of the results being available	43 (84)
Between 11–30 days from the results being available	4 (8)
30 days or more	1 (2)
Results are Not Returned	1 (2)
Do not remember	1 (2)
No Response	1 (2)
**Spirometry**	
Within 10 days of the results being available	36 (70)
Between 11–30 days from the results being available	4 (8)
30 days or more	0 (0)
Results are Not Returned	9 (18)
Do not remember	1 (2)
No Response	1 (2)
**Do you feel participants want to receive their research results?**	
Yes	47 (92)
No	1 (2)
Don’t Know	2 (4)
No Response	1 (2)
**Do you feel you have answers to participants’ questions about their results?**	
Always	11 (21)
Often	25 (49)
Sometimes	10 (20)
Occasionally	3 (6)
Never	1 (2)
No Response	1 (2)
Comfort returning research results by Role** **Mean (SD) [range]**	
Coordinator	3.8 (1.1) [2–5]
Investigator	4.4 (1.0) [1–5]
Other (e.g., Research Nurse)	4.4 (0.5) [4–5]

* 26 represents the number of research sites

** Likert Scale 1–5 (1 = Not at all comfortable, 5 = Very Comfortable)

**Table 2:** Open-Ended Survey Responses (N = 45)

Theme	Description	Quote from Participants
**Q12.** Describe any BENEFITS to returning research results to LHC participants.		
Early Detection & Health Awareness	Returning results enables early identification of possible health issues, giving participants valuable insights into their health status.	*“Participants often would not have had access to a CT scan otherwise, and this allows them to identify potential issues earlier.”* (Survey Respondent 28)
Recruitment, Engagement & Retention	Sharing results strengthens participant engagement and trust, motivating ongoing involvement in the research study.	*“Many subjects participate in research to have the tests to better understand their health. It is a recruitment tool and a retention tool to share their results with them if they are standard of care testing.”* (Survey Respondent 42)
Access to Valuable Health Information	Providing results allows access to exams and health information (e.g., CT scans, spirometry, lab tests) that participants might not otherwise receive.	*“Some participants show more engagement knowing they’ll receive results they would not normally have from annual PCP visits like spirometry.”* (Survey Respondent 20)
Building Trust & Transparency	Returning results builds trust and transparency between researchers and participants, enhancing the collaborative nature of the research experience.	*“I feel like returning the results helps build a better relationship with our participants. Along with building trust, it helps keep participants engaged…”* (Survey Respondent 33)
**Q13.** Describe any CHALLENGES experienced with returning LHC research results to participants.		
Communication Barriers	Difficulty reaching participants by phone, email, or EHR messages.	*“Multiple contacts required. EHR messages may not be read.”* (Survey Respondent 34)
Health Literacy & Interpretation	Many participants do not understand their results, particularly incidental/ambiguous findings.	*“Even when results are shared, some participants may lack the health literacy to interpret the data, requiring more time and effort from the research team to address concerns.”* (Survey Respondent 28)
Follow-up Care Complications	Lack of primary care or insurance can hinder participants’ ability to act on abnormal results or seek recommended care.	*“Sometimes abnormal finding requires follow up labs or CT. Some pts have insurance coverage, others don’t so it can create difficulty in finding them appropriate access to the follow up needed.”* (Survey Respondent 5)
Increased Research Team Workload	Requires multiple outreach attempts, thorough explanation, and addressing anxiety, increasing demand on team resources.	*“Returning results can increase the workload for the principal investigator (PI) and the research team… many still turn to the PI with questions, requiring additional time to explain results or offer guidance. This not only diverts focus from the study’s research objectives but also places a strain on the team.”* (Survey Respondent 48)
Balancing Disclosure & Clinical Value	Dilemmas in deciding what to disclose, especially findings of uncertain significance; concern about causing unnecessary worry or leading to overtreatment.	*“We don’t know the true meaning of some of our results (mosaic attenuation) and so it is difficult to assign importance and is complicated to talk about whether it is significant or not.”* (Survey Respondent 10)
**Q14.** How can you be better SUPPORTED in returning results to LHC participants?		
Standardized Communication Tools & Templates	Use IRB-approved templates and standard language for consistency when sharing results with participants.	*“If there are templates available for relaying these results to participants, it would be helpful. Study coordinators often find it challenging to communicate certain type of medical results so having talking points would be great!”* (Survey Respondent 3)
Enhanced Educational Resources	Provide educational, user-friendly materials to help participants understand results and recommendations for next steps.	*“Providing dedicated resources, such as informational guides or access to healthcare navigators, could help participants… understand how to address abnormal results.”* (Survey Respondent 42)
Improved Protocols & Guidelines	Set clear, consistent policies and guidelines for returning results and managing incidental findings.	*“Receiving clearer and more direct guidelines with what results we can/cannot share with LHC participants would be helpful.”* (Survey Respondent 37)
Additional Clinical Support	Increase access to clinical staff for delivering sensitive news and supporting participant follow-up.	*“More physician time dedicated to helping return results and answer questions…”* (Survey Respondent 32)
Centralized & Streamlined Processes	Standardize and potentially centralize certain elements to ensure efficiency and consistency across sites.	*“Centralized safety reads would be helpful to ensure all participants at all sites are receiving consistent feedback.”* (Survey Respondent 40)

**Table 3:** Barriers and Potential Solutions

CFIR Level	Barriers	Potential Solutions
**Innovation**	Multiple communication formats causing inconsistency; confusion around “abnormal but not clinically significant” findings; unclear interpretation of incidental findings; participant anxiety when results arrive before the team can follow-up.	Standardized templates, scripts, and patient-friendly summaries; clear diagnostic definitions; written documentation for both participants and PCPs; timely coordination so the team can support participants before or alongside result delivery.
**Outer Setting**	Participants vary in technology access and preferences (EHR, phone, mail, in-person); limited access to follow-up care or PCPs; community health centers not always linked to main hospitals.	Flexible, site-adaptable communication methods (phone, in-person, portal, mail); demographic-appropriate resources; strengthen referral pathways to community health providers and navigators.
**Inner Setting**	Workflow delays from radiology and data management; lack of clarity on who communicates results; multiple team members involved causing confusion; institutional policy variation across sites; operational inefficiencies with outside data.	Define clear roles and responsibilities; streamline radiology/data workflows; clarify protocol in the Manual of Procedures (MOP); enhance coordinator documentation; improve data integration systems (e.g., EHR tools, technology supports).
**Individuals: Roles & Characteristics**	Coordinators and non-clinical members feel unprepared to explain complex results; discomfort communicating incidental findings; workload burden for PIs and coordinators; limited training.	Provide training, mentoring, and decision-support tools; designate clinician support (NPs, physicians, fellows) for complex results; create a trained “results specialist” role with allocated time.
**Implementation Process**	Lack of standardization in communication workflows; delayed follow-up increases anxiety; debate over centralized vs site-level result return (efficiency vs personal rapport and trust); privacy and PHI transfer concerns.	Develop standardized but customizable communication workflows; implement monitoring and feedback loops; balance centralized efficiencies with local site relationships; establish secure PHI/data transfer processes; educate participants on result return procedures.

**Table 4:** Vignette A

Vignette A: Current State-Research Coordinator Doing Their Best in a Complex System
Maria, a research coordinator with three years of experience but no formal clinical training, begins her day at 8:15 a.m. by opening the results queue in Redcap. She reviews three chest CT reports: two normal and one categorized as “abnormal but not clinically significant” a designation she immediately recognizes as challenging to communicate.
She consults the study protocol, prior documentation, and recent team correspondence but finds no standardized guidance for explaining this type of result. Instead, she must develop her communication approach in real time.
Maria begins outreach. One call goes to voicemail, another number is disconnected, and a third participant answers but is unaware of the reason for contact, having not accessed the patient portal. Maria reports the imaging result.
The participant asks, “Do I have cancer?” Maria carefully balances transparency with reassurance, explaining that the result came back abnormal but that does not mean cancer. She continues by saying, “it would be best to discuss this with your provider who knows you and your health best.” The participant reports not having a provider and asks if he should go to the emergency department, shifting the interaction from result disclosure to care navigation.
Throughout the day, Maria manages competing responsibilities: tracking outreach attempts, responding to participant messages.
Later, a participant contacts the team after independently viewing results in the patient portal, expressing concern about an “abnormal” finding. Maria provides clarification, but only after anxiety has already been triggered in the participant.
By the end of the day, Maria has made repeated outreach attempts and addressed a wide range of participant questions. Although returning results is viewed as valuable, persistent gaps in guidance, workflow standardization, and support remain. Communication depends heavily on Maria’s ability to interpret, translate, and reassure in real time, contributing to both cognitive and emotional burden.

**Table 4:** Vignette B

Vignette B: Future State-A Coordinated, Participant-Centered Return of Results System
At 8:15 a.m., Maria logs into an integrated results dashboard. New chest CT results are automatically categorized (normal, non-urgent finding, clinically actionable), each linked to a standardized communication pathway.
Maria selects a “non-urgent finding.” A participant-facing summary is generated, including plain-language explanation, visual risk context, and recommended next steps. A role-specific script provides key messaging and anticipated questions.
During the call, Maria uses the script as a guide to explain that the finding is common, low-risk, and does not require immediate follow-up. When the participant asks about concern, Maria responds confidently using standardized, reassuring language.
When the participant reports not having a primary care provider, Maria provides a referral list and connects the participant to a patient navigator, ensuring clear next steps.
Between calls, Maria reviews the tracking system, which documents outreach status, confirms result delivery, and flags participants requiring follow-up. Automated alerts support timely communication, eliminating uncertainty.
Participant messaging reflects improved understanding because results are delivered alongside clear explanations and next steps.
For complex cases, Maria follows a defined escalation pathway, engaging clinical support for additional consultation. Responsibilities are clearly delineated, and workflows are consistent across sites.
By the end of the day, Maria completed result outreach, answered questions, and supported participants within a structured system. Standardized tools, defined roles, and integrated supports reduce individual burden and enhance communication quality.

## Data Availability

The datasets generated and/or analyzed during the current study are not publicly available due to the inclusion of potentially identifiable research team information but may be are available from the corresponding author on reasonable request.

## Abbreviations

ACRC: Airways Clinical Research Centers
ALA: American Lung Association
CFIR: Consolidated Framework for Implementation Research
CT: Computed Tomography
EHR: Electronic Health Record
IRB: Institutional Review Board
IRR(s): Individual Research Result(s)
LHC: Lung Health Cohort
MOP: Manual of Procedures
NHLBI: National Heart, Lung, and Blood Institute
PCP: Primary Care Provider
PI: Principal Investigator
REDCap: Research Electronic Data Capture
RECEIVE: Returning Computed Tomography Results to Active Lung Health Cohort Participants
SRQR: Standards for Reporting Qualitative Research
